# Intraoperative lactate level correlated with the length of hospital stay in patients with intraoperative hypothermia

**DOI:** 10.1097/MD.0000000000043168

**Published:** 2025-07-04

**Authors:** Qin Xu, Chen-Lu Hu, Hai-Yan Xiang, Jian-Wen Yang, Wei-Ming Qian, Jian-Ping Song

**Affiliations:** a Nursing Department, The Second Affiliated Hospital Zhejiang University School of Medicine, Hangzhou, Zhejiang Province, China.

**Keywords:** hypothermia, lactate, length of hospital stay, Medical Information Mart for Intensive Care-IV

## Abstract

This study aims to explore potential biomarkers correlated with the clinical outcome only in patients with intraoperative hypothermia undergoing cardiac surgery. The clinical data of patients with or without intraoperative hypothermia, and 8 blood indicators both in preoperative and intraoperative periods, were obtained from the Medical Information Mart for Intensive Care-IV database. The associations of indicators with the length of hospital stay (LOS) were explored in non-hypothermia and hypothermia groups, respectively. The key indicator showing the association only in hypothermia groups was selected for further analysis. The detailed association between the key indicator and LOS was further determined by a series of analyses, including restricted cubic spline, generalized additive model, generalized linear regression, trend regression, threshold effects analysis, and quartile regression. Their clinical value was explored by the receiver operating characteristic analysis and decision curve analysis. Among 16 indicators, only the intraoperative lactate was identified to correlate with the LOS in patients with hypothermia rather than normal group (all *P* < .05). Lactate showed a positive and nonlinear association with LOS (all *P* < .05), and their association was especially observed when log-lactate > 2.420 (all *P* < .05). Quartile regression showed that the associations of lactate with LOS quartiles from 40% and 80% were stably increased when log-lactate > 2.420 (all *P* < .05). However, no association was observed in any analyses when log-lactate ≤ 2.420. We also confirmed the favorable prediction performance and clinical net benefit of lactate on the LOS. Intraoperative lactate is significantly associated with the LOS of patients with hypothermia and is a useful biomarker.

## 1. Introduction

Therapeutic hypothermia has been commonly regarded as a neuroprotective measure during cardiac surgeries, mainly because therapeutic hypothermia reduces the cerebral metabolic rate and energy depletion, decreases excitatory transmitter release, inhibits apoptosis, and reduces the free radical generation.^[1]^ However, hypothermia, at the same time, can significantly impact various physiological functions of the body. For example, it can inhibit the chemotaxis of neutrophils, the activity of macrophages, and the function of the complement system, weakening the anti-infectious ability.^[2]^ In addition, hypothermia also inhibits Na^+^/K^+^-ATP activity, leading to potassium ion efflux from cells, which may induce hypokalemia and trigger arrhythmia.^[3]^ It has been found that hypothermia was significantly correlated with several unfavorable clinical outcomes including surgical site infections, myocardial complications, electrolyte depletion, postoperative delirium, prolonged recovery from anesthesia, and increased length of hospital stay (LOS).^[4–6]^ Perioperative hypothermia is a common phenomenon with an incidence ranging from 20 to 70%.^[7]^ In addition, the requirement of therapeutic hypothermia was often considered necessary during cardiac surgeries. It follows that the prediction of hypothermia, especially among patients undergoing cardiac surgery, has limited value. Therefore, the identification of populations with poor clinical outcomes is more significant and necessary in these patients who experienced intraoperative hypothermia, which contributes to improving the patient’s clinical outcome.

Currently, few researchers have reported the indicators that predicted the clinical outcome, especially in patients who experienced intraoperative hypothermia.^[8]^ Pasquier et al found that age, sex, and serum potassium level were associated with the survival to hospital discharge of patients who suffered cardiac arrest due to accidental hypothermia.^[9]^ However, it is unclear whether serum potassium level was specifically related to the outcome only in patients undergoing accidental hypothermia. Several common risk factors, such as age, gender, and comorbidity, are non-modifiable factors. The application of therapeutic hypothermia is even more indispensable during cardiac surgical treatment. Therefore, focusing on these non-modifiable factors is of little value. It is more important to identify more modifiable factors that can effectively predict the clinical outcome in patients undergoing cardiac surgery, especially in preoperative and intraoperative periods, but not postoperative.^[10,11]^

The collections of laboratory blood indicators are inexpensive and convenient; however, their important predicted value is always ignored. In this study, we obtained some preoperative and intraoperative blood indicators of patients undergoing cardiac surgery and paid more attention to identifying the key indicator that was related to the clinical outcome only in patients with hypothermia.

## 2. Methods

### 2.1. Participants

All the data were from the Medical Information Mart for Intensive Care-IV database. Medical Information Mart for Intensive Care-IV is a publicly available single-center critical care database. All patient-related information was anonymous, and informed consent was not required. Inclusion criteria: age range from 18 to 89; received the cardiac surgery; had a record of the intraoperative temperature; for patients with multiple admissions records, we only retained the record of the patient’s first admission; no deaths occurred during hospitalization. Exclusion criteria: the LOS < 2 days; intraoperative temperature > 38.3℃; record duplication; obvious error about the value of temperature data.

### 2.2. Data collection

In this study, the baseline characteristics of patients included gender (male, female), age (≤55, >55 yr), marital status (single, married, divorced, widowed), obesity (non-obese, obese), and comorbidities including hypertension, diabetes, peripheral vascular disease, and hyperlipidemia. We also collected several blood indicators both in preoperative and intraoperative periods, including monocyte (%), lymphocyte (%), red cell distribution width (RDW, %), uric acid (mg/dL), creatinine (mg/dL), anion gap (AG, mEq/L), lactate (IU/L), and urea nitrogen (mg/dL). In this study, the primary clinical outcome was the LOS (d) and in-hospital death (yes, no).

### 2.3. Statistical analysis

The blood variables associated with the LOS were initially explored by the univariable generalized linear regression in non-hypothermia and hypothermia groups, respectively. Then, the key blood indicators associated with the LOS in the hypothermia group but not in the non-hypothermia group were selected for further analysis. We subsequently analyzed the differences in terms of baseline characteristics, comorbidities, and key blood indicators between LOS ≤ 7 days and LOS > 7 days groups only in patients with intraoperative hypothermia. Hypothermia is defined by a body core temperature below 36.0°C.^[7]^ The categorical variables were expressed as frequency (%), and their distribution differences between the 2 groups were analyzed by *χ*^2^ test. Continuous variables with normal distribution were expressed as mean and standard deviation, and differences between 2 groups were analyzed by *t* test. The data with abnormal distribution was expressed as median and quartile, and differences between the 2 groups were analyzed by the Mann–Whitney *U* test. The clinical value of key blood indicators on the LOS of patients with hypothermia was explored by the receiver operating characteristic (ROC) analysis and decision curve analysis (DCA). The association of key indicators with the LOS was assessed by restricted cubic spline (RCS), generalized additive model, generalized linear regression, and trend regression analyses. Threshold effects analysis was conducted to explore the important turning point affecting their association. Quartile regression analysis was performed to evaluate the association changes of key indicators with the different quartiles of LOS. Finally, ROC and DCA analyses were also conducted to evaluate the clinical value of key indicators on the in-hospital death. All the analysis was performed by SPSS version 20.0 (IBM Corp., Armonk) and R software version 3.6 (R Foundation for Statistical Computing, developed at the University of Auckland, Auckland, New Zealand), and *P* < .05 was considered statistically significant.

## 3. Results

### 3.1. The differential indicators identification in patients with hypothermia

**Figure 1.**
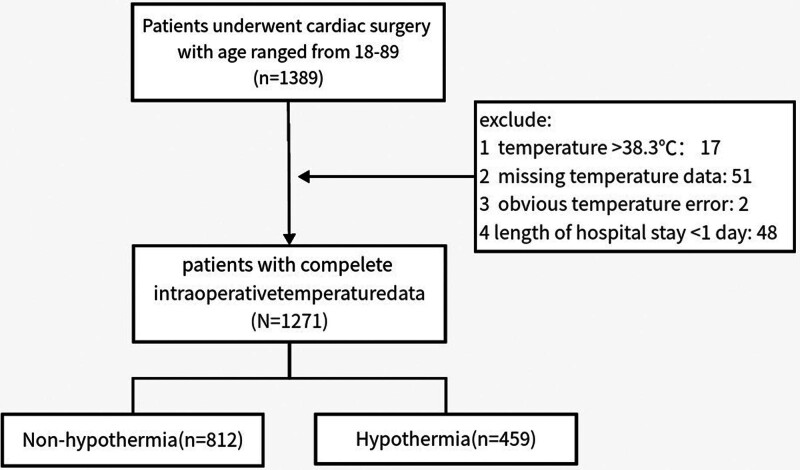
The flowchart of participants selection in this study.

This study totally enrolled 1271 patients in the analysis, including 812 non-hypothermia patients and 459 hypothermia patients. The flowchart of participants selection was presented in Figure 1. We first explored the association of 8 blood indicators in preoperative and intraoperative periods with the LOS in non-hypothermia and hypothermia groups, respectively. The results in Table 1 showed that preoperative creatinine, RDW, lymphocyte, AG, and lactate, as well as intraoperative RDW and lymphocyte, were significantly associated with the LOS both in non-hypothermia and hypothermia patients (all *P* < .05). Preoperative and intraoperative urea nitrogen, as well as intraoperative creatinine, monocyte, and AG all showed no association with LOS (all *P* > .05) in separate 2 groups. Especially, the levels of uric acid in preoperative and intraoperative periods, preoperative monocyte, and intraoperative lactate all showed significant associations with LOS just in patients with hypothermia (all *P* < .05), but their association was insignificant in patients without hypothermia (all *P* > .05). These 4 indicators were regarded as the differential indicators only associated with the clinical outcome of patients with hypothermia. Therefore, our study further explored the clinical value of these indicators only in patients with hypothermia. Due to the small sample size of uric acid, the preoperative monocyte and intraoperative lactate were selected for further analysis. Due to the high discrete degree of intraoperative lactate, we transformed the actual lactate value as log-lactate.

**Table 1:** The association between blood indicators and hospital LOS in patients grouped by hypothermia.

Variables	Non-hypothermia (n = 812)			Hypothermia (n = 459)		
	N	*β* (95%CI)	*P* value	N	*β* (95%CI)	*P* value
Preoperative indicators						
Creatinine (mg/dL)	812	0.806 [0.496–1.116]	<.001	459	1.146 [0.554–1.738]	<.001
Urea nitrogen (mg/dL)	220	−0.003 [−0.007–0.000]	.088	91	0.001 [−0.006–0.008]	.814
Uric acid (mg/dL)	87	−0.22 7 [−0.714–0.260]	.361	33	0.555 [0.113–0.997]	.014
RDW (%)	812	0.527 [0.293–0.760]	<.001	458	0.859 [0.485–1.233]	<.001
Lymphocyte (%)	426	−0.132 [−0.195–−0.070]	<.001	200	−0.100 [−0.182–−0.018]	.017
Monocyte (%)	428	−0.116 [−0.380–0.148]	.388	200	0.418 [0.110–0.726]	.008
Anion gap (mEq/L)	812	0.288 [0.171–0.406]	<.001	459	0.426 [0.258–0.595]	<.001
Lactate (IU/L)	493	0.001 [0.000–0.002]	.047	258	0.003 [0.002–0.004]	<.001
Intraoperative indicators						
Creatinine (mg/dL)	80	0.304 [−0.554–1.162]	.487	31	0.485 [−0.158–1.127]	.139
Urea nitrogen (mg/dL)	194	−0.003 [−0.007–0.001]	.216	83	0.000 [−0.008–0.008]	.993
Uric acid (mg/dL)	80	−0.262 [−0.688–0.165]	.229	31	0.513 [0.096–0.930]	.016
RDW (%)	796	0.529 [0.267–0.791]	<.001	449	0.949 [0.487–1.411]	<.001
Lymphocyte (%)	654	−0.123 [−0.168–−0.078]	<.001	390	−0.242 [−0.31–−0.174]	<.001
Monocyte (%)	654	−0.086 [−0.278–0.106]	.381	390	−0.061 [−0.342–0.220]	.670
Anion gap (mEq/L)	806	0.011 [−0.159–0.180]	.902	457	−0.113 [−0.316–0.089]	.272
Lactate (IU/L)	442	0.001 [−0.002–0.001]	.769	240	0.002 [0.001–0.003]	<.001

CI = confidence interval, LOS = length of hospital stay, RDW = red cell distribution width.

Then all the patients with hypothermia were divided into 2 groups according to the hospital LOS (≤7 vs > 7 d). The baseline characteristics of patients are presented in Table 2. Between the 2 groups, the distributions of hyperlipidemia, hypertension, and peripheral vascular comorbidities were all different (all *P* < .05). APS-III scores, Charlson comorbidity index, and in-hospital death rate were all different between the 2 groups (all *P* < .05). In terms of preoperative monocyte and intraoperative log-lactate levels, only lactate levels showed significant differences between 2 groups (*P* < .001). Therefore, we finally selected intraoperative lactate as the research topic in this study.

**Table 2:** The baseline characteristics of patients with hypothermia grouped by hospital LOS.

Variables	Subgroups	LOS ≤ 7 d (n = 390)	LOS > 7 d (n = 69)	*P* value
Gender	Male	240 (61.538)	40 (57.971)	.575
	Female	150 (38.462)	29 (42.029)	
Age	≤55 yr	42 (10.769)	13 (18.841)	.057
	>55 yr	348 (89.231)	56 (81.159)	
Marital status	Single	66 (17.694)	10 (15.873)	.486
	Married	214 (57.373)	36 (57.143)	
	Divorced	37 (9.920)	10 (15.873)	
	Widowed	56 (15.013)	7 (11.111)	
Obesity	Non-obese	102 (62.577)	16 (59.259)	.742
	Obese	61 (37.423)	11 (40.741)	
Hyperlipidemia	No	156 (40.000)	46 (66.667)	<.001
	Yes	234 (60.000)	23 (33.333)	
Diabetes	No	272 (69.744)	46 (66.667)	.610
	Yes	118 (30.256)	23 (33.333)	
Hypertension	No	149 (38.205)	40 (57.971)	.002
	Yes	241 (61.795)	29 (42.029)	
Peripheral vascular	No	363 (93.077)	59 (85.507)	.033
	Yes	27 (6.923)	10 (14.493)	
Age		71 [62, 77]	70 [60, 77]	.465
Preoperative monocyte		5.6 [4.2, 7.0]	6.4 [4.8, 8.7]	.064
Intraoperative log-lactate		2.394 [2.292, 2.539]	2.577 [2.433, 2.955]	<.001
APS-III		36 [28, 50]	71 [50, 92]	<.001
CCI		5 [4, 7]	7 [5, 9]	<.001
In-hospital death	No	374 (95.897)	62 (89.855)	.034
	Yes	16 (4.103)	7 (10.145)	

Among 459 patients with hypothermia, 453 patients showed mild hypothermia (33–36℃).

CCI = Charlson comorbidity index, LOS = length of hospital stay.

### 3.2. The association between intraoperative lactate and hospital LOS in patients with hypothermia

**Figure 2.**
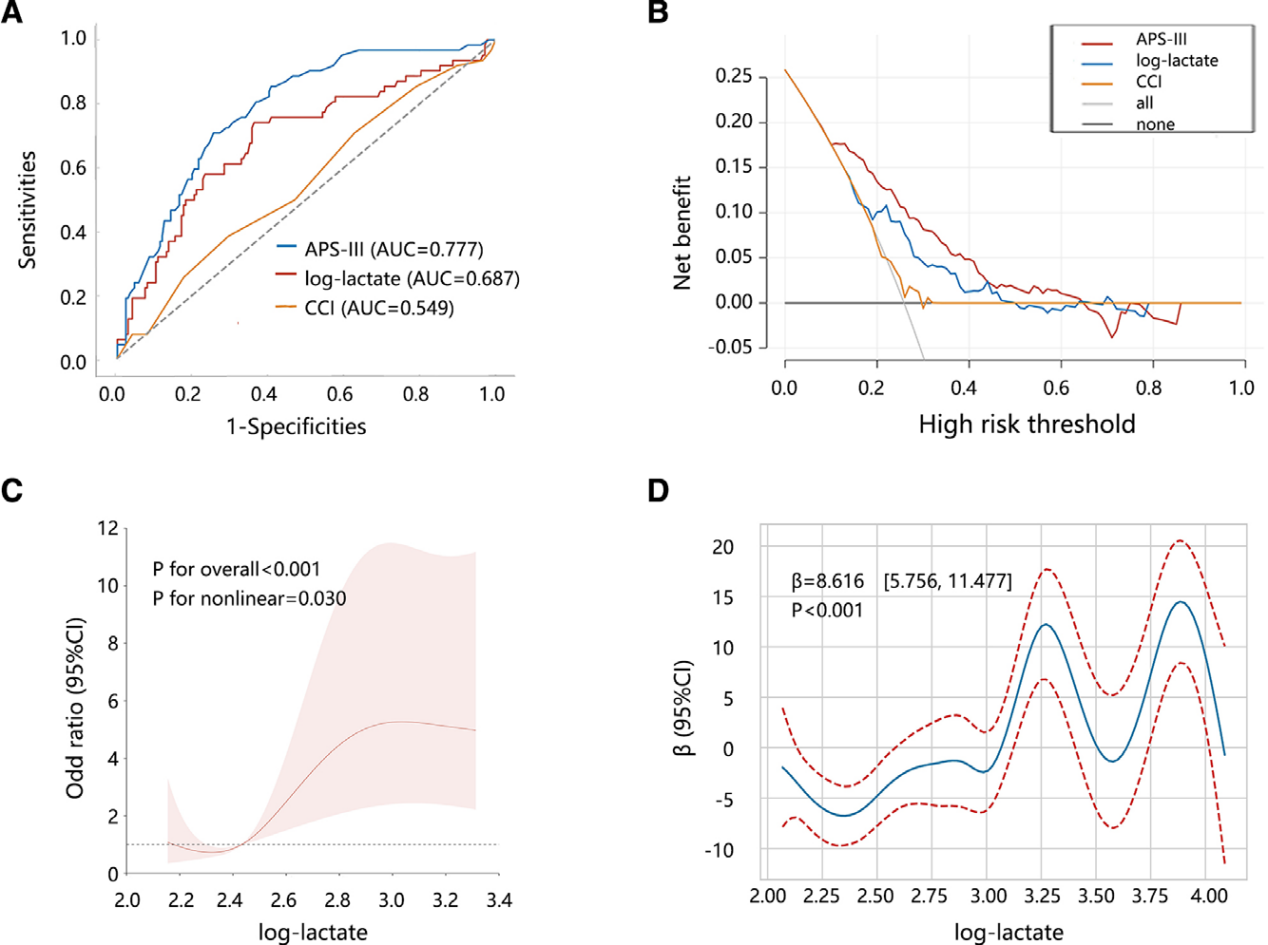
The clinical value of intraoperative lactate in the hospital LOS of patients with hypothermia. (A) Prediction performance assessment by ROC analysis. Delong test: APS-III vs CCI (*P* < .001); log-lactate vs CCI (*P* = .021); APS-III vs log-lactate (*P* = .072). (B) Clinical net benefit analysis by DCA analysis. (C) Correlation analysis between lactate and 7-day LOS by RCS analysis. (D) Correlation analysis between lactate and LOS by GAM analysis. DCA = decision curve analysis, GAM = generalized additive model, LOS = length of hospital stay, RCS = restricted cubic spline, ROC = receiver operating characteristic.

According to the above results, intraoperative lactate was considered a key indicator associated with the clinical outcome of patients with hypothermia. We then evaluated the clinical value of intraoperative lactate in the clinical outcome of patients with hypothermia by comparing it with the APS-III and CCI, as APS-III^[12]^ and CCI^[13]^ have been confirmed as important predictors of LOS. The ROC analysis (Fig. 2A) showed a prediction performance of intraoperative lactate on the 7-day LOS with an AUC of 0.687 (95%CI: 0.606–0.768), which was superior to CCI (AUC = 0.549, 95%CI: 0.498–0.620, Delong test *P* = .021) but similar with APS-III (AUC = 0.777, 95%CI: 0.602–0.835, Delong test *P* = .072). DCA analysis indicated that intraoperative lactate achieved a favorable clinical net benefit (Fig. 2B). ROC and DCA analyses suggested the potential clinical value of intraoperative lactate in patients with hypothermia. Then, RCS (Fig. 2C, *P* = .030) and generalized additive model (Fig. 2D, *P* < .001) analyses all supported a nonlinear correlation between intraoperative lactate and hospital LOS of patients.

Due to the nonlinear correlation between intraoperative lactate and hospital LOS of patients, generalized linear regression analysis was then conducted to evaluate their association. The results (Table 3) showed that continuous log-lactate level was significantly associated with the hospital LOS of patients in Model 1 (*β* = 8.516), Model 2 (*β* = 6.162), and Model 3 (*β* = 6.079). Even though the *β* was decreased with the increase of adjusted variables, their association was consistently significant in 3 models (all *P* < .001). We then explored the changes in their association, finding that with the increase of log-lactate quartiles, their association was significantly increased among the 3 models (all P for trend < 0.05). These results suggested their significant association.

**Table 3:** The association analysis between intraoperative lactate and hospital LOS in patients with hypothermia after adjusting for different variables.

	Model 1 *β* (95%CI)	Model 2 *β* (95%CI)	Model 3 *β* (95%CI)
log-lactate	8.616 [5.756–11.477] ***	6.162 [3.348–8.976] ***	6.079 [3.234–8.925] ***
Quartiles			
Q1 [2.068–2.309]	Reference	Reference	Reference
Q2 [2.310–2.431]	−0.510 [−3.482–2.462]	−0.844 [−3.567–1.879]	−1.473 [−4.251–1.305]
Q3 [2.432–2.604]	1.525 [−1.434–4.484]	−0.217 [−3.027–2.594]	−0.667 [−3.574–2.239]
Q4 [2.605–4.088]	6.747 [3.788–9.706] ***	4.490 [1.520–7.460] **	3.833 [0.788–6.879] *
*P* for trend	<.001	.010	.027

Model 1: no adjustment.

Model 2: with adjustment of age, gender, marital status, and obesity.

Model 3: with adjustment of age, gender, marital status, obesity, hyperlipidemia, diabetes, hypertension, and peripheral vascular comorbidities.

LOS = length of hospital stay.

*P < .05; **P < .01, ***P < .001.

The above RCS analysis suggested their nonlinear correlation, and trend regression analysis found that their association was mainly found in the Q4 group (all *P* < .05 in 3 Models). These results indicated that there may be a key point that affected their association. Therefore, a threshold effect analysis was then conducted to identify the key point.

The results of threshold effect analysis (Table 4) indicated that the LRT tests both in crude and full-adjusted models were all significant, which further confirmed the nonlinear association between intraoperative lactate and hospital LOS of patients. From the threshold effect analysis, a significant turning point of log-lactate = 2.420 was found. On the left of the turning point, their association was not significant. However, their association was significant on the right of the turning point (*β* = 9.092, *P* = .001).

**Table 4:** The threshold effects analysis on intraoperative lactate in patients with hypothermia.

	Crude model *β* (95%CI)	Full-adjusted model *β* (95%CI)
Model 1		
One-line effect	8.616 [5.756–11.477], <0.001	6.079 [3.234–8.925], <0.001
Model 2		
Turning point (K)	log-lactate = 2.253	log-lactate = 2.420
<K	−35.037 [−69.530–−0.543], 0.047	−10.534 [−22.219–1.152], 0.080
>K	9.998 [6.959–13.017], 0.013	9.092 [5.658–12.526], 0.001
P for LRT	0.013	0.003

The full-adjusted model included the adjustment of age, gender, marital status, obesity, hyperlipidemia, diabetes, hypertension, and peripheral vascular comorbidities.

**Figure 3.**
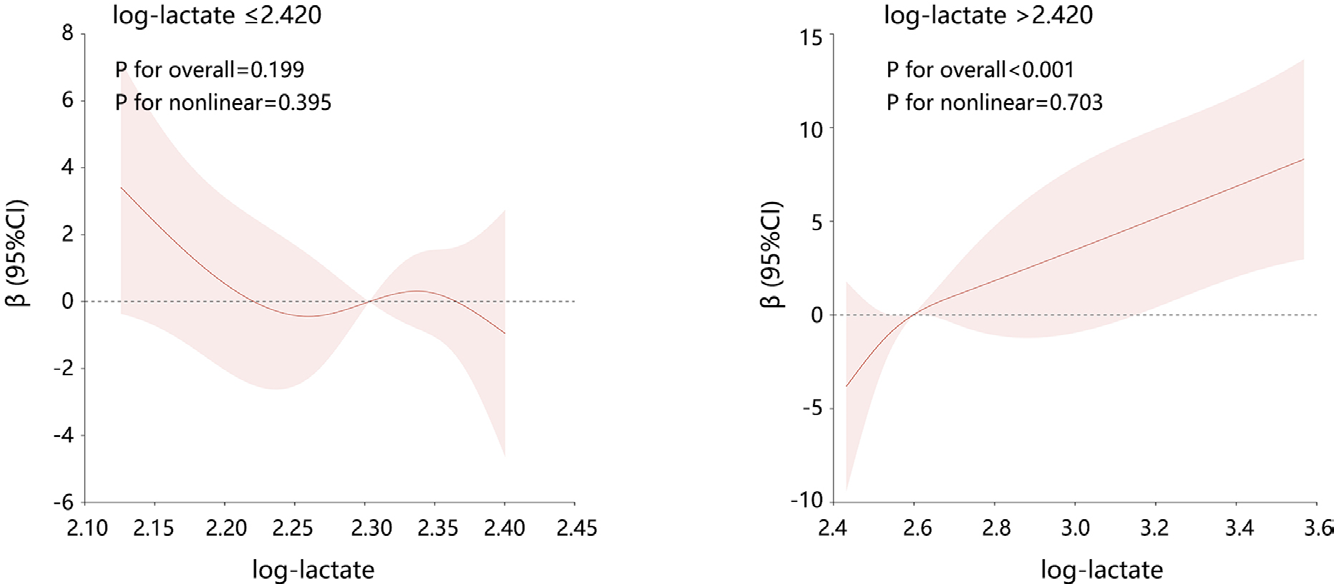
The association between intraoperative lactate and hospital LOS in patients with log-lactate ≤ 2.420 and log-lactate > 2.420, respectively. LOS = length of hospital stay.

According to the turning point, all the patients were divided into 2 clusters, and their correlation was detected again (Fig. 3). Among the 2 clusters, the correlations between intraoperative lactate and hospital LOS were all linear (all P for nonlinear > 0.05). However, their significant association was just found on the right of the turning point (*P* for overall < .001). These results were in line with the results of threshold effect analysis.

We further performed a quartile regression to explore the association of intraoperative lactate with different quartiles of hospital LOS grouped by a turning point of 2.420 (Table 5). The results showed that intraoperative lactate showed no significant association with any of the quartiles of hospital LOS in log-lactate ≤ 2.420 group (all *P* < .05), which implied the validity of turning point as 2.420. Among patients with log-lactate > 2.420, their significant association was first found in the 40% quartiles of hospital LOS (LOS = 3.18 days, *β* = 4.398, *P* = .003). With the increase of hospital LOS quartiles, their association was significantly increased (50–90% quartiles: *β* from 7.664 to 29.588, all *P* < .001).

**Table 5:** The association between intraoperative lactate and hospital LOS by quartile regression.

Quartiles (d)	log-lactate ≤ 2.420 [*β* (95%CI), *P* value]	log-lactate > 2.420 [*β* (95%CI), *P* value]
0.1	−0.333 [−3.893–3.227], 0.853	1.176 [−0.930–3.283], 0.271
0.2	−1.348 [−5.411–2.714], 0.512	2.251 [−0.236–4.738], 0.076
0.3	−1.543 [−5.904–2.819], 0.485	1.983 [−0.647–4.613], 0.138
0.4	−0.198 [−4.98–4.586], 0.935	4.398 [1.541–7.253], 0.003
0.5	−3.119 [−8.480–2.241], 0.251	7.664 [4.497–10.830], <0.001
0.6	−0.739 [−7.163–5.685], 0.820	11.110 [7.709–14.511], <0.001
0.7	−2.914 [−10.362–4.535], 0.440	12.484 [8.264–16.704], <0.001
0.8	−0.662 [−16.251–14.927], 0.933	15.166 [8.880–21.450], <0.001
0.9	−26.279 [−52.881–0.324], 0.053	29.588 [19.116–40.059], <0.001

LOS = length of hospital stay.

**Figure 4.**
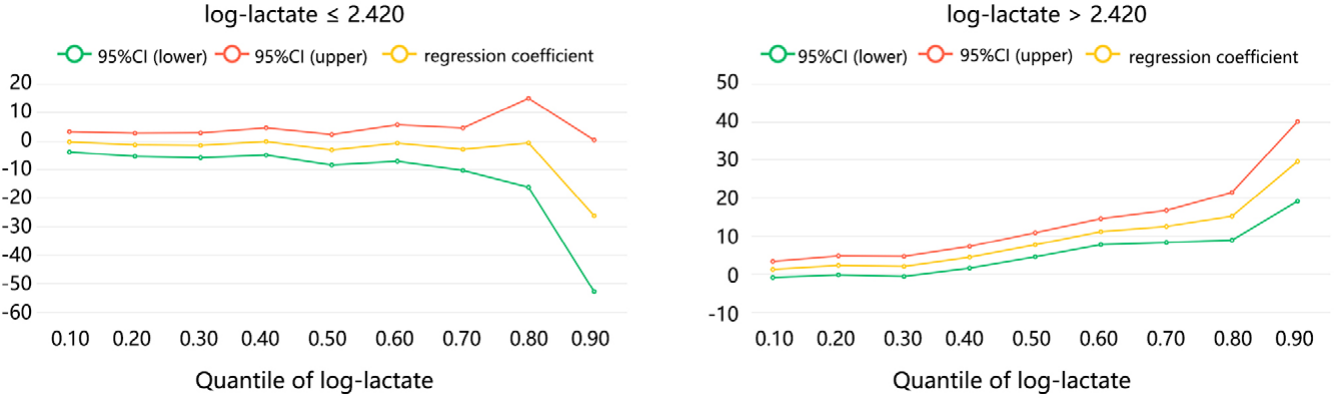
The association between intraoperative lactate and hospital LOS of patients with hypothermia by quartile regression grouped by log-lactate turning point of 2.420. LOS = length of hospital stay.

The quartile regression results were also visualized in Figure 4. Among the patients with log-lactate > 2.420, the changes in correlation coefficient can be found in 3 zones. There were almost no changes in the 10–30% LOS quartile. A trend of stable linear increase can be found in the 40–80% quartile (LOS from 3.18 to 12.61 days). A sharp increase was found in the 90% quartile. It followed that when the hospital LOS was located at 3.18 to 12.61 days, the association of intraoperative lactate and hospital LOS was significant, with a stable increase.

### 3.3. The value assessment of intraoperative lactate in-hospital death

**Figure 5.**
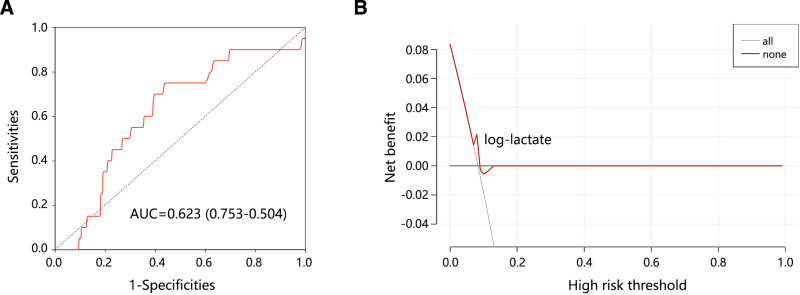
The clinical value of intraoperative lactate in the in-hospital death of patients with hypothermia. (A) Performance of intraoperative lactate for predicting in-hospital death by ROC analysis. (B) Clinical net benefit obtained by DCA analysis. DCA = decision curve analysis, ROC = receiver operating characteristic.

Finally, we explored the clinical value of intraoperative lactate in in-hospital death. Although the ROC analysis showed the potential value of intraoperative lactate in predicting in-hospital death (Fig. 5A, AUC = 0.623), the DCA analysis showed an unsatisfactory clinical net benefit (Fig. 5B). These results highlighted the importance of intraoperative lactate in the LOS of patients with hypothermia.

## 4. Discussion

In this study, we initially collected 8 blood indicators both in preoperative and intraoperative periods, finding that preoperative monocyte and intraoperative lactate levels were related to the LOS only in patients with hypothermia (rather than in the normal group). Further, only intraoperative lactate showed the difference between LOS ≤ 7 and LOS > 7 days groups. This study first identified a key indicator correlated only with the clinical outcome of patients with hypothermia.

We further found that intraoperative lactate had important clinical value in predicting the hospital LOS of patients with hypothermia, and it was significantly related to the LOS in a positive association. The association between intraoperative lactate and the clinical outcome of patients undergoing cardiac surgery has been revealed. The previous studies have indicated that the increase of intraoperative lactate level was associated with a higher mortality in low- and medium-risk procedures^[14]^ and longer duration of ICU stay^[15]^ in pediatric cardiac surgery. Duval B found that intraoperative changes in blood lactate levels were associated with overall ICU morbidity and 30-day all-cause mortality after cardiac surgery with cardiopulmonary bypass.^[16]^ These results all highlighted the importance of intraoperative lactate levels on the clinical outcome of patients undergoing cardiac surgery. However, an important factor, the required intraoperative hypothermia during cardiac surgery, was not taken into consideration in previous studies. Currently, no study revealed whether hypothermia can change their association in the population with intraoperative hypothermia. In this study, we found that the association between intraoperative lactate level and LOS was only observed in the hypothermia group rather than the normal group. It follows that the association assessment between intraoperative hypothermia and clinical outcomes in patients undergoing cardiac surgery should not disregard hypothermia. This finding may not be extrapolated to the non-hypothermia patients. The previous study suggested that although the correlation between intraoperative lactate levels and LOS was more pronounced in hypothermic conditions, this correlation was not significant in the general population at normal body temperature.^[17]^ This may be due to the impact of hypothermia on metabolic processes, making changes in lactate levels better reflect the patient’s physiological state and postoperative recovery. Hypothermia can reduce the hepatic blood flow and mitochondrial oxidative phosphorylation efficiency, leading to decreased lactate clearance.^[18]^ In addition, by activating the sympathetic nervous system, hypothermia induces peripheral vasoconstriction, which diminishes tissue perfusion and increases anaerobic metabolism, thereby elevating lactate production.^[19]^ Additionally, hypothermia decreases the glomerular filtration rate,^[20]^ delaying renal excretion of lactate. These mechanisms collectively contribute to the elevated blood lactate levels. Consequently, under hypothermic conditions, increased lactate levels may indicate more severe organ hypoperfusion, inflammatory responses, or metabolic compensation imbalance, all of which prolong hospitalization. In contrast, normothermic patients exhibit stable lactate levels, as their lactate metabolic pathways remain largely unimpaired. The absence of correlation between lactate levels and LOS in this group suggested milder metabolic stress and faster recovery. In addition, we also found that intraoperative lactate had a poor clinical net benefit for predicting in-hospital death. This result may suggest the limited value of intraoperative lactate for predicting in-hospital death, especially in patients with hypothermia.

Serum lactate levels are a useful marker of the imbalance between oxygen supply and demand resulting from circulatory impairment.^[21]^ Excessive lactate is often produced due to tissue hypoxia during anaerobic metabolism.^[22]^ During cardiac surgery, patients are always faced with an increased risk of anemia-induced tissue hypoxia.^[23]^ It has been reported that erythropoietin, but not methemoglobin, is a potential biomarker of anemia-induced tissue hypoxia.^[24]^ After cardiac surgery, hyperlactatemia is common due to anaerobic metabolism.^[25]^ The increase in serum lactate levels during routine cardiac surgery was also related to the intraoperative decrease in lactate clearance.^[26]^ In addition, hypoperfusion is the main contributor to the increase in lactate.^[27]^ Different cerebral perfusion techniques on top of hypothermia have been applied to avoid any period of intraoperative brain ischemia.^[27]^ Lactate as the metabolite of anaerobic metabolism has been widely used as the ICU biomarker, as well as it has been incorporated into multiple management algorithms.^[28]^

Lactate is not only a metabolite of cellular metabolism, but also acts as a complex immunomodulatory molecule.^[29]^ The advantageous effects of lactate in the period of acute inflammation have been discussed. However, lactate also mediates immunosuppression and influences the immune function of immune cells.^[30]^ Lactate can promote chronic inflammation mainly by driving T cells’ immune dysfunction,^[31]^ such as inhibiting the T cell’s migratory capability^[32]^ and causing the loss of cytolytic activity of CD4^+^ and CD8^+^ T cells.^[33]^ The loss of motility makes T cells entrapped at the inflamed sites and increases the production of inflammatory cytokines, thus leading to chronic inflammation.^[34]^ In this study, we found that higher intraoperative lactate level was significantly associated with longer LOS of patients with hypothermia, which was unfavorable for the patient’s clinical outcome. The poor prognosis associated with the high lactate may be due to the chronic inflammation effect. The persistent inflammation immunosuppression caused longer hospital stays, worse prognosis, and more adverse outcomes.^[35]^

This study reveals that intraoperative lactate is an important biomarker and positively associated with the LOS of patients with hypothermia. Our study suggested that intraoperative lactate is a useful biomarker for assessing the short outcome of patients. In addition, we should pay great attention to the increase of intraoperative lactate during the operation, indicating that the internal environment is extremely disordered, which may be caused by shock. Doctors will attach great importance to actively replenishing blood volume, dealing with symptoms, and treating the cause. In addition, an increase in intraoperative lactate may imply the abnormality of cardiac function, respiratory function, as well as liver and kidney function. Therefore, it is of great clinical significance to pay close attention to lactic acid level during operation.

Finally, several limitations should be stated. In this study, almost all the patients with hypothermia refer to mild hypothermia (33–36℃). It is uncertain whether other hypothermia categories will affect their association. In addition, only 240 samples have the intraoperative lactate record among patients with hypothermia. Research-based larger sample sizes may be better and more persuasive. Moreover, this study was a retrospective single-center study; therefore, the scientific verifications using single-center or multicenter cohorts in the future are needed for our findings to be more convincing.

## 5. Conclusion

Among 8 blood indicators, both in preoperative and intraoperative periods, the intraoperative lactate was identified to correlate with the hospital LOS only in patients with hypothermia (not in the normal group). With the increase of intraoperative lactate, the hospital LOS significantly increased. When the LOS was located at 3.18 to 12.61 days, the association between intraoperative lactate and hospital LOS was significant, with a stable increase. Intraoperative lactate not only had favorable prediction performance on the hospital LOS but also achieved a better clinical net benefit. Intraoperative lactate is an important biomarker among patients with hypothermia.

## Author contributions

**Conceptualization:** Qin Xu.

**Data curation:** Qin Xu, Hai-Yan Xiang, Jian-Ping Song.

**Formal analysis:** Qin Xu, Jian-Wen Yang.

**Investigation:** Jian-Wen Yang, Wei-Ming Qian.

**Methodology:** Chen-Lu Hu, Wei-Ming Qian.

**Supervision:** Jian-Ping Song.

**Writing – original draft:** Qin Xu, Chen-Lu Hu, Hai-Yan Xiang, Jian-Wen Yang, Wei-Ming Qian, Jian-Ping Song.

**Writing – review & editing:** Wei-Ming Qian, Jian-Ping Song.

## References

[1] Otto K. Therapeutic hypothermia applicable to cardiac surgery. Vet Anaesth Analg. 2015;42:559–69. 26361886 10.1111/vaa.12299

[2] Najder K Rugi M Lebel M. Role of the intracellular sodium homeostasis in chemotaxis of activated murine neutrophils. Front Immunol. 2020;11:2124. 33013896 10.3389/fimmu.2020.02124 PMC7506047

[3] Boubes K Batlle D Tang T. Serum potassium changes during hypothermia and rewarming: a case series and hypothesis on the mechanism. Clin Kidney J. 2023;16:827–34. 37151414 10.1093/ckj/sfac158 PMC10157793

[4] Lau A Lowlaavar N Cooke E. Effect of preoperative warming on intraoperative hypothermia: a randomized-controlled trial. Can J Anaesth. 2018;65:1029–40. 29872966 10.1007/s12630-018-1161-8

[5] Akers J Dupnick A Hillman E Bauer A Kinker L Wonder A H. Inadvertent perioperative hypothermia risks and postoperative complications: a retrospective study. AORN J. 2019;109:741–7. 31135987 10.1002/aorn.12696

[6] Ju J Nam K Sohn J. Association between intraoperative body temperature and postoperative delirium: a retrospective observational study. J Clin Anesth. 2023;87:111107. 36924749 10.1016/j.jclinane.2023.111107

[7] Ruetzler K Kurz A. Consequences of perioperative hypothermia. Handb Clin Neurol. 2018;157:687–97. 30459033 10.1016/B978-0-444-64074-1.00041-0

[8] Takada K Nagamine Y Ishii A. Association between intraoperative early warning score and mortality and in-hospital stay in lower gastrointestinal spontaneous perforation. Anesthesiol Res Pract. 2023;2023:8910198. 37674585 10.1155/2023/8910198 PMC10480023

[9] Pasquier M Hugli O Paal P. Hypothermia outcome prediction after extracorporeal life support for hypothermic cardiac arrest patients: the HOPE score. Resuscitation. 2018;126:58–64. 29481910 10.1016/j.resuscitation.2018.02.026

[10] Devlin J Skrobik Y Gélinas C. Clinical practice guidelines for the prevention and management of pain, agitation/sedation, delirium, immobility, and sleep disruption in adult patients in the ICU. Crit Care Med. 2018;46:e825–73. 30113379 10.1097/CCM.0000000000003299

[11] Aldecoa C Bettelli G Bilotta F. European Society of Anaesthesiology evidence-based and consensus-based guideline on postoperative delirium. Eur J Anaesthesiol. 2017;34:192–214. 28187050 10.1097/EJA.0000000000000594

[12] Granholm A Christiansen C Christensen S Perner A Møller M. Performance of SAPS II according to ICU length of stay: a Danish nationwide cohort study. Acta Anaesthesiol Scand. 2019;63:1200–9. 31197823 10.1111/aas.13415

[13] Liu H Song B Jin J. Length of stay, hospital costs and mortality associated with comorbidity according to the charlson comorbidity index in immobile patients after ischemic stroke in China: a national study. Int J Health Policy Manag. 2022;11:1780–7. 34380205 10.34172/ijhpm.2021.79 PMC9808248

[14] Alves R Aragão e Silva A Kraychete N Campos G Martins MJ Módolo N. Intraoperative lactate levels and postoperative complications of pediatric cardiac surgery. Paediatr Anaesth. 2012;22:812–7. 22409574 10.1111/j.1460-9592.2012.03823.x

[15] Kanazawa T Egi M Shimizu K Toda Y Iwasaki T Morimatsu H. Intraoperative change of lactate level is associated with postoperative outcomes in pediatric cardiac surgery patients: retrospective observational study. BMC Anesthesiol. 2015;15:29. 25759606 10.1186/s12871-015-0007-y PMC4354761

[16] Duval B Besnard T Mion S. Intraoperative changes in blood lactate levels are associated with worse short-term outcomes after cardiac surgery with cardiopulmonary bypass. Perfusion. 2019;34:640–50. 31250726 10.1177/0267659119855857

[17] Andersen LW Holmberg MJ Doherty M. Postoperative lactate levels and hospital length of stay after cardiac surgery. J Cardiothorac Vasc Anesth. 2015;29:1454–60. 26456273 10.1053/j.jvca.2015.06.007

[18] Minor T Stegemann J Hirner A Koetting M. Impaired autophagic clearance after cold preservation of fatty livers correlates with tissue necrosis upon reperfusion and is reversed by hypothermic reconditioning. Liver Transpl. 2009;15:798–805. 19562717 10.1002/lt.21751

[19] Lee YS Kim WY Yoo JW Jung HD Min TJ. Correlation between regional tissue perfusion saturation and lactate level during cardiopulmonary bypass. Korean J Anesthesiol. 2018;71:361–7. 29690753 10.4097/kja.d.17.00002 PMC6193595

[20] Deferm N Annink KV Faelens R. Glomerular filtration rate in asphyxiated neonates under therapeutic whole-body hypothermia, quantified by mannitol clearance. Clin Pharmacokinet. 2021;60:897–906. 33611729 10.1007/s40262-021-00991-6 PMC8249265

[21] Sugita S Ishikawa M Sakuma T Iizuka M Hanai S Sakamoto A. Intraoperative serum lactate levels as a prognostic predictor of outcome for emergency abdominal surgery: a retrospective study. BMC Surg. 2023;23:162. 37328824 10.1186/s12893-023-02075-7 PMC10276372

[22] Cata J Bhavsar S Hagan K. Intraoperative serum lactate is not a predictor of survival after glioblastoma surgery. J Clin Neurosci. 2017;43:224–8. 28601568 10.1016/j.jocn.2017.05.004

[23] Shehata N Mazer C. Red cell transfusion in cardiac surgery: what is the right balance? Transfusion. 2019;59:903–4. 30776088 10.1111/trf.15200

[24] Hare G Han K Leshchyshyn Y. Potential biomarkers of tissue hypoxia during acute hemodilutional anemia in cardiac surgery: a prospective study to assess tissue hypoxia as a mechanism of organ injury. Can J Anaesth. 2018;65:901–13. 29696581 10.1007/s12630-018-1140-0

[25] Piot J Hébrard A Durand M Payen J Albaladejo P. An elevated respiratory quotient predicts complications after cardiac surgery under extracorporeal circulation: an observational pilot study. J Clin Monit Comput. 2019;33:145–53. 29667097 10.1007/s10877-018-0137-0

[26] Ganushchak Y Maessen J de Jong D. The oxygen debt during routine cardiac surgery: illusion or reality? Perfusion. 2002;17:167–73. 12017383 10.1191/0267659102pf561oa

[27] Zymliński R Biegus J Sokolski M. Increased blood lactate is prevalent and identifies poor prognosis in patients with acute heart failure without overt peripheral hypoperfusion. Eur J Heart Fail. 2018;20:1011–8. 29431284 10.1002/ejhf.1156

[28] Englert J Rogers A. Metabolism, metabolomics, and nutritional support of patients with sepsis. Clin Chest Med. 2016;37:321–31. 27229648 10.1016/j.ccm.2016.01.011 PMC5084839

[29] Manoharan I Prasad P Thangaraju M Manicassamy S. Lactate-dependent regulation of immune responses by dendritic cells and macrophages. Front Immunol. 2021;12:691134. 34394085 10.3389/fimmu.2021.691134 PMC8358770

[30] Zhang Y Zhai Z Duan J. Lactate: The mediator of metabolism and immunosuppression. Front Endocrinol. 2022;13:901495. 10.3389/fendo.2022.901495 PMC9218951 35757394

[31] Pucino V Certo M Bulusu V. Lactate buildup at the site of chronic inflammation promotes disease by inducing CD4 T cell metabolic rewiring. Cell Metab. 2019;30:1055–74.e8. 31708446 10.1016/j.cmet.2019.10.004 PMC6899510

[32] Gerriets V Rathmell J. Metabolic pathways in T cell fate and function. Trends Immunol. 2012;33:168–73. 22342741 10.1016/j.it.2012.01.010 PMC3319512

[33] Haas R Marelli-Berg F Mauro C. In the eye of the storm: T cell behavior in the inflammatory microenvironment. Am J Clin Exp Immunol. 2013;2:146–55. 23885332 PMC3714175

[34] Luo Y Li L Chen X Gou H Yan K Xu Y. Effects of lactate in immunosuppression and inflammation: progress and prospects. Int Rev Immunol. 2022;41:19–29. 34486916 10.1080/08830185.2021.1974856

[35] Zhou Q Qian H Yang A Lu J Liu J. Clinical and prognostic features of chronic critical illness/persistent inflammation immunosuppression and catabolism patients: a prospective observational clinical study. Shock. 2023;59:5–11. 36383370 10.1097/SHK.0000000000002035

